# Long-Term Effects of Adjuvant Intravitreal Treatment with Autologous Bone Marrow-Derived Lineage-Negative Cells in Retinitis Pigmentosa

**DOI:** 10.1155/2021/6631921

**Published:** 2021-06-02

**Authors:** Marta P. Wiącek, Wojciech Gosławski, Aleksandra Grabowicz, Anna Sobuś, Miłosz P. Kawa, Bartłomiej Baumert, Edyta Paczkowska, Sławomir Milczarek, Bogumiła Osękowska, Krzysztof Safranow, Alicja Zawiślak, Wojciech Lubiński, Bogusław Machaliński, Anna Machalińska

**Affiliations:** ^1^First Department of Ophthalmology, Pomeranian Medical University, Powst. Wlkp. 72, 70-111 Szczecin, Poland; ^2^Second Department of Ophthalmology, Pomeranian Medical University, Powst. Wlkp. 72, 70-111 Szczecin, Poland; ^3^Department of General Pathology, Pomeranian Medical University, Powst. Wlkp. 72, 70-111 Szczecin, Poland; ^4^Department of Bone Marrow Transplantation, Department of Hematology and Bone Marrow Transplantation, Pomeranian Medical University, Unii Lubelskiej 1, 71-252 Szczecin, Poland; ^5^Department of Biochemistry and Medical Chemistry, Pomeranian Medical University, Powst. Wlkp. 72, 70-111 Szczecin, Poland

## Abstract

**Background:**

Autologous bone marrow-derived lineage-negative (Lin−) cells present antiapoptotic and neuroprotective activity. The aim of the study was to evaluate the safety and efficacy of novel autologous Lin− cell therapy during a 12-month follow-up period.

**Methods:**

Intravitreal injection of Lin− cells in 30 eyes with retinitis pigmentosa (RP) was performed. The fellow eyes (FEs) were considered control eyes. Functional and morphological eye examinations were performed before and 1, 3, 6, 9, and 12 months after the injection.

**Results:**

Patients whose symptoms started less than 10 years ago gained 14 ± 10 letters, while those with a longer disease duration gained 2.86 ± 8.54 letters compared to baseline at the 12-month follow-up (*p* = 0.021). There were significantly higher differences in response densities of *P*1-wave amplitudes in the first ring of multifocal ERGs in treated eyes than FE recordings in all follow-up points were detected. Accordingly, the mean deviation in 10-2 static perimetry improved significantly in the treated eyes compared with fellow eyes 12 months after the procedure. The QoL scores improved significantly and lasted until the 9-month visit.

**Conclusion:**

Lin− cell-based therapy is safe and effective, especially for a well-selected group of RP patients who still maintained good function of the foveal cones.

## 1. Introduction

Retinitis pigmentosa (RP) is the most frequent form of retinal degeneration with a prevalence of 1 : 4000. The essential problems leading to significant visual impairment in RP are both, progressive loss of the peripheral visual field and impaired night vision caused by deterioration of peripheral cones, and rod function. As the disease progresses, the macula is also affected, causing the deterioration of far and near visual acuity, photophobia, loss of colour vision, and complete blindness in the advanced stage. The characteristic symptoms of macular involvement include macular atrophy, oedema, and epiretinal membrane formation [1].

More than 3000 mutations in over 200 genes responsible for this RP-like clinical picture have been detected to date [2]. The age of onset and progression rate is strictly associated with the inheritance type and patient's genotype [1, 2]. The products of mutated genes are proteins responsible for structure, function, metabolism, and other biochemical processes in retinal cells [3]. However, the influence of environmental factors might also have an impact on the course of RP. The rich blood supply and high metabolic rate in the presence of decreased demand by destroyed rods in the retina might be potential causes of cone dysfunction due to oxidative stress. On the other hand, the uveal volume is decreased due to deteriorated blood flow in this area in RP. Other factors potentially exaggerating retinal function in RP are increased expression of microglia and proinflammatory cytokines secondary to extrabulbar inflammatory processes [1, 4–6].

Objective evaluation of retinal function with full-field electroretinography (ERG) is essential for the diagnosis. In RP, abnormal scotopic electrical activity primarily manifesting as a decreased amplitude, prolonged peak time, and photopic ERG is detected. As the disease progresses, further deterioration of the response until complete absence of the signal is observed. The recommended examination for detection of residual function and monitoring in advanced cases of RP is multifocal electroretinography (mfERG). The foveal part of the retina in RP is characterized by well-preserved responses, while the mean peripheral responses are significantly reduced, and the peak times are longer [1]. In the first two decades of life, the progression of RP is slow and mildly symptomatic, but in patients older than 20 years who have the autosomal recessive form and in those older than 32 years who have the autosomal dominant form, 10-20% loss of visual field per year is observed. As a result, loss of function accelerates almost exponentially [1, 3].

Several methods have been tested for therapeutic efficacy in RP in an attempt to limit the pathophysiological processes involved in the aetiology of RP. Some vitamins are important cofactors for metabolic processes and have an antioxidative effect. However, in RP, dietary supplementation with vitamin A, lutein, and docosahexaenoic acid even in multiplied doses led to no significant improvement in retinal function. In contrast, vitamin E intake was related to a more rapid decline in ERG amplitudes. Moreover, preliminary results of a few studies testing the anti-inflammatory activity of valproic acid were not encouraging enough to pursue further investigation. The replacement of mutated genes in damaged parts of the retina delivered by viral vectors is an interesting treatment modality. However, several issues, such as numerous inheritance patterns, safety concerns, targeting, and low cargo capacity of the vector or necessity for an invasive administration route during pars plana vitrectomy, are problematic in RP patients. These methods presented acceptable safety levels but poor efficacy; thus, the idea of neuroprotective treatment in RP seems to be a promising direction for further research [7]. The neuroprotective effect was evaluated after an intraocular implant with retinal pigment epithelium (RPE) cells transfected with the human ciliary neurotrophic factor (CNTF) gene due to CNTF production. On the other hand, the administration of stem and progenitor cells (SPCs) to individuals with RP is expected to result in trophic support for the host's photoreceptors, especially the cones that are still alive. Neuroprotective factors released by SPCs such as “classical” neurotrophins (NTs) including brain-derived factor (BDNF), neurotrophin-3 (NT-3), and neurotrophin-4 (NT-4) may serve as neurotransmitter modulators to augment the secretion of other deficient neurotrophic protective factors. We previously reported that human-derived lineage-negative (Lin−) cells enriched in immature SPCs abundantly express several NTs including BDNF, NGF, NT-3, NT-4, and angiogenic cytokines such as vascular endothelial growth factor (VEGF) [8]. Importantly, NT expression in the Lin− cell population was stronger than that in unsorted bone-marrow-derived nucleated cells (NCs) [8]. We have also demonstrated that these secreted factors support neuronal survival in a conditioned medium from a Lin− population [8]. The influence of intravitreal administration of Lin− cells on retinal morphology and function in a mouse model of retinal injury was the subject of our previously published study [9]. We observed that the antiapoptotic and neuroprotective activities of Lin− cells were related to a significant increase in local BDNF expression. Specifically, the paracrine properties of Lin− cells injected into the eye allow for the continuous secretion of neurotrophic and immunomodulatory factors [9]. Indeed, we found that cells injected as therapy into the vitreous cavity of Rd6 mutant mice, used as a model for chronic retinal degeneration, survived at least 3 months [10]. Moreover, such cells preferentially integrated into the retinal layers, which could facilitate specific targeting to the neuroretina and considerably rescue damaged retinal cells, as evaluated by ERG and immunofluorescence staining [10]. Considering such characteristics, neuroprotection via cell-based neurotrophic therapies offers a new all-encompassing approach for chronic retinal photoreceptor pathologies.

In this study, we investigated the clinical and morphological changes in eyes subjected to a single intravitreal injection of autologous Lin− cells in RP patients. The aim of the study was to evaluate the safety and efficacy of implemented cell therapy during the 12-month follow-up period. In particular, we hypothesized that adjuvant cell-based therapy could yield a specific benefit to the biological status of cone photoreceptors as they remained alive despite the loss of rod photoreceptor function.

## 2. Materials and Methods

### 2.1. Subjects

This prospective, open-label, nonrandomized, single center clinical trial was approved by the institutional review board and adhered to the tenets of the Declaration of Helsinki. Moreover, the study was registered with identifying number NCT03772938 as a stem cell clinical trial at the National Institutes of Health (NIH) at https://pubmed.ncbi.nlm.nih.gov/21863221/. The National Health Ministry approvals for cell acquisition, preparation, and distribution were also obtained.

**Figure 1:**
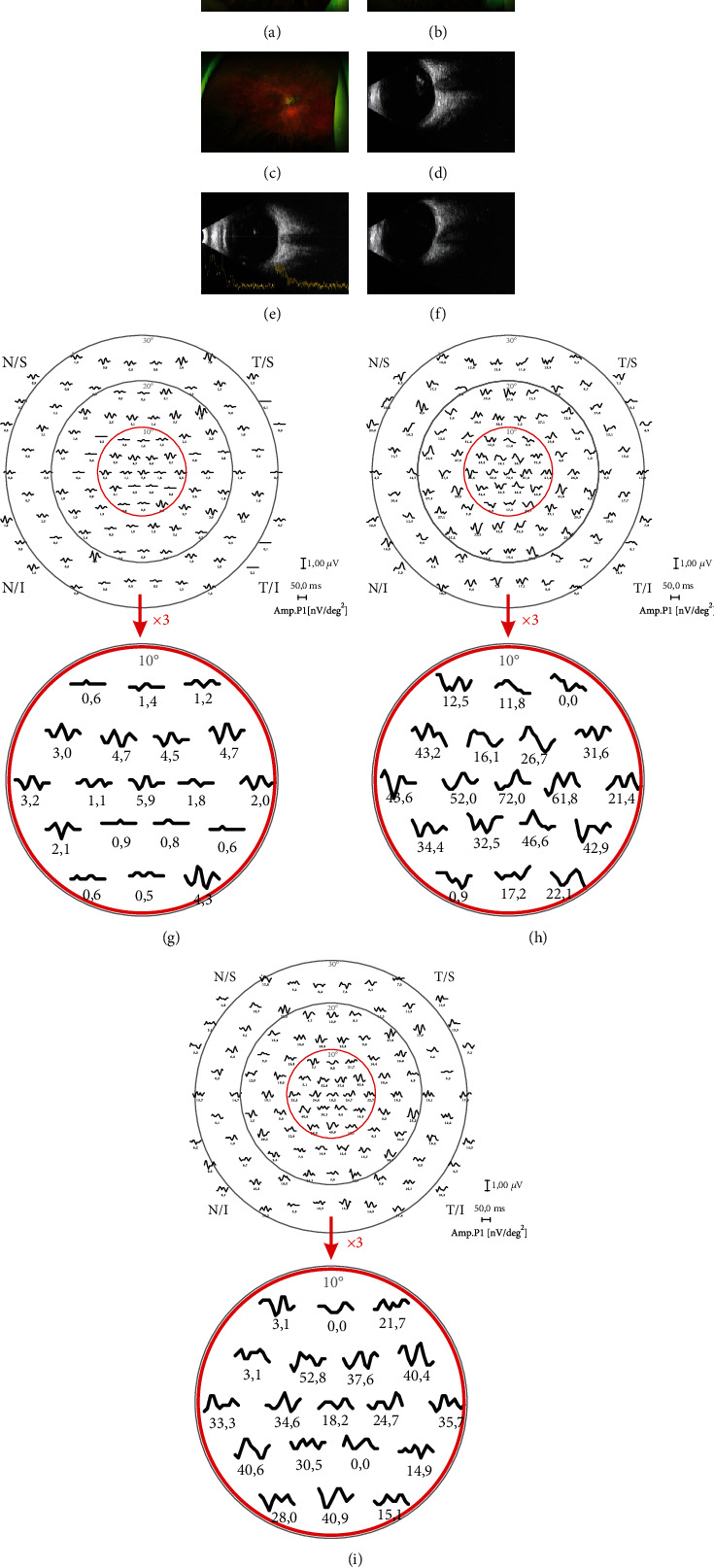
Representative results of the patient with retinitis pigmentosa treated with intravitreal injection of autologous bone marrow-derived lineage-negative cells: eye fundus image recorded (a) directly after injection, (b) 1 month, and (c) 12 months postinjection; globe sonography (d) directly after injection, (e) 1 month, and (f) 12 months postinjection; and multifocal electroretinography (mfERG) recordings presented as amplitudes of P1 wave with highlighted central 10° at (g) baseline, (h) 1 month, and (i) 12 months postinjection.

Prior to the intervention, all subjects were assessed by the qualifying committee, which was formed by ophthalmology, internal medicine, transplantation, and hematology specialists. Each patient's current general condition was established based on a detailed physical examination, chest X-rays, abdominal US, ECG, and blood biochemistry and inflammatory marker tests. Considering each patient's overall systemic condition, blood tests, and ophthalmological condition, the qualifying committee decided to enrol the patient for the procedure of bone marrow aspiration for the isolation of Lin− cells, which were administered by intravitreal injection. As a result, 30 patients with RP (12 females and 18 males) with a mean age of 41.72 ± 12.77 (range: 19 and 64) years were enrolled for the study. The clinical diagnosis of RP was made based on the following criteria: characteristic fundus with a waxy pallor of the optic disc (Figure 1(a)), decreased diameter of the retinal vessels, and intraretinal pigment in the midperiphery; history of progressive impairment of night or colour vision, loss of peripheral vision, photophobia, decreased visual acuity, and prolonged dark adaptation; and peripheral constriction up to “tunnel vision” on a visual field examination with significant reduction of amplitudes with prolonged implicit times or unrecordable readings in electroretinography in ERG [1]. According to the patients' declarations, in 9 subjects, symptoms had started less than 10 years prior, and in 21 individuals, their symptoms had lasted for over 10 years. In all individuals, genetic testing with the whole-exome sequencing (WES) method was performed. The patients' genetic characteristics are presented in Table 1. In 4 patients, the genetic analysis did not reveal the mutation responsible for the clinical image of RP. The analysis is still ongoing. In those individuals, the decision of enrolment for the study was made on the basis of the clinical appearance of the disease. The following inclusion criteria must have been met together: clinical diagnosis of RP, age 18–65 years, BCDVA in the worse eye subjected to therapy within range 0.1 to 0.5 Snellen letter chart, and ability to express written informed consent. Any other eye diseases, i.e., significant cataracts, glaucoma, retinopathy, eye trauma, or ophthalmological surgery within 3 months, systemic disease (acute inflammatory or autoimmunological process, recent trauma, renal or hepatic failure, thyroid disease, cardiovascular or neurological disease, stroke, neoplasms, and DM), intake of medications or other diseases that could affect the cells' effect, dietary supplementation or any other RP treatment during the course of the study, and previously applied stem cell-based therapies were defined as exclusion criteria.

### 2.2. Bone Marrow Collection and Isolation of Lin– Cells

Bone marrow (BM) was aspirated with local anaesthesia from the posterior iliac crest. Prior to the intervention, all patients gave their informed consent for the procedure. The volume of obtained BM together with the anticoagulating solution (heparin and saline) ranged from 48 to 149 ml. All the following steps of cell isolation were performed in accordance with good manufacturing practice (GMP) in a closed isolator system Xvivo (BioSperix, Ltd., Parish, NY, USA). A mononucleated cell suspension was obtained via centrifugation in lymphocyte separation medium (MP Biomedicals, Santa Ana, CA, USA). In the next step, the negative isolation of Lin– cells was performed using the commercially available Lineage Cell Depletion Kit (Miltenyi Biotec, Auburn, AL, USA), as described previously [8]. Briefly, the cells were mixed with biotin-conjugated monoclonal antibodies against antigens present on the surface of lineage-positive cells (CD2, CD3, CD11b, CD14, CD15, CD16, CD19, CD56, CD123, and CD235a) and antibiotin monoclonal antibodies bound to microbeads. Then, they were passed through magnetic columns. The obtained population of unlabelled Lin– cells was washed and suspended in sterile phosphate-buffered saline (Thermo Fisher Scientific, Waltham, MA, USA).

### 2.3. Intravitreal Injection Procedure

A single administration of Lin− cell suspension to eyes with more advanced stages of disease defined as a lower BCDVA was performed. The intravitreal injection was performed in an operating room. Preparation of the eye included the instilling of local anaesthetic drops. Prior to injection, the area of skin around the eye, eyelids, and eyelashes was washed with a 10% solution of povidone iodine. After placing a sterile drape, the conjunctival sac was rinsed additionally with a 5% solution of povidone iodine. After 90 seconds and rinsing the eye with physiological saline, depending on the lens status, the intravitreal injection was performed at a distance of 3.5 to 4.0 mm from the limbus of the cornea in the inferotemporal quadrant. Prior to the injection, the Lin− cell suspension was centrifuged. The number of administered Lin− cells was 10^6^ suspended in 0.05 mL of phosphate-buffered saline. As a prevention of early intraocular pressure (IOP) spike, the paracenthesis with auqeous humour release was performed as a standard procedure.

### 2.4. Assessment of the Local Effect and Safety of the Therapeutic Intervention

In order to improve the vigilance in peri-interventional period, each patient was admitted to ophthalmology department and released 5 days after the procedure. To ensure the safety of the Lin− cell treatment, additional evaluation on the 7th day with a detailed examination of vision, IOP, as well as anterior and posterior segment of the treated eye was performed. However, the follow-up examination used in statistical analysis was based on complete ophthalmological examination of both eyes performed before Lin− cell injection at 1, 3, 6, 9, and 12 months after the procedure. The fellow eye (FE) was considered as a sham arm. The examinations included measurements of distant (BCDVA) and near (BCNVA) best-corrected visual acuity measured on an ETDRS chart for BCDVA and a Snellen chart for BCNVA, contrast sensitivity (Pelli-Robson chart), IOP (dynamic contour tonometry, SMT Swiss Microtechnology AG, Port, Switzerland), retinal optical coherence tomography (OCT, Heidelberg Engineering, Heidelberg, Germany), 10-2 and 30-2 (W-W) static perimetry (HFA, Zeiss, Dublin, CA), and a slit lamp examination. Particular emphasis was placed on the maintenance of the same lighting conditions during all follow-up examinations. The eye fundus ultra-widefield imaging (Optos, Marlborough, MA, USA) and globe sonography (Accutome Inc., Malvern, Pennsylvania) were performed due to monitor cell dispersion in the vitreous cavity (Figures 1(a)–1(f)).

In all follow-up examinations, patients underwent electrophysiological examinations in the following order due to pupil diameter requirements: pattern electroretinography (PERG, RetiScan system, Roland Consult, Germany), multifocal electroretinography (mfERG, RetiScan system, Roland Consult, Germany), and full-field electroretinography (ERG, UTAS-E 2000, LKC Technologies, Gaithersburg, Maryland, United States). Recordings were obtained according to the International Society for Clinical Electrophysiology of Vision (ISCEV) standards [11–13].

The PERG recordings were obtained unilaterally using a black and white reversing checkerboard displayed on a computer CRT monitor with parameters set according to the standards of Pomeranian Medical University (PMU) laboratory [14]. The check size was set at 1°, the luminance of the white elements was equal to 120 cd/m^2^, the contrast between black and white squares (Michelson contrast) was set to 97%, and the temporal frequency was set at 4.0 rps (2.0 Hz). The following parameters of the recording system were used: amplifier sensitivity: 20 *μ*V/div; filter frequency bandwidth: 1–100 Hz; notch filters: off; sweep time: 250 ms (time base, 25 ms/div); and artefact reject threshold: 95% (for the amplifiers range ± 100 *μ*V); and averaging: 200 waveforms. The patient was placed in front of the monitor with the eyes fixed at central point, and the pupils did not require dilation. A proper spectacle correction for an eye-screen distance of 0.5 metres was used if needed. A thread Dawson–Trick–Litzkow (DTL) electrode (Diagnosys LLC, Lowell, Massachusetts, United States) was defined as an active electrode. One gold disc skin electrode (Roland Consult, Brandenburg, Germany) was placed in lateral canthus on the same side and was defined as a reference electrode. A similar electrode was attached in the middle of the forehead and served as a ground electrode. The stimulation was repeated twice, and then the two waveforms were averaged in the postacquisition time. The amplitude (*μ*V) and peak time (ms) of the P50 wave as well as the amplitude (*μ*V) of the N95 wave were analysed.

The preparation for mfERG included maximal dilation of the pupil with 10% phenylephrine hydrochloride and spectacle correction for a 0.3-meter distance. Unilateral stimulation with a black and white matrix of 103 hexagons with a distortion factor set at 4 was performed. Central fixation was used. The matrix covered 30° of center to edge from the displayed fixation point. A luminance was equal to 100 cd/m^2^, and a Michelson contrast of 97% was applied. A thread DTL electrode (Diagnosys LLC, Lowell, Massachusetts, United States) was used as an active electrode. A gold disc skin electrode (Roland Consult, Brandenburg, Germany) placed at the ipsilateral outer canthus served as a reference electrode. A similar electrode was attached in the middle of the forehead (Fpz) and acted as a ground electrode. To reduce random bias, each eye was tested six times, and the results were averaged. Additionally, both automatic double postacquisition smoothing and line interference reduction as well as manual correction of the cursor location were performed. The following parameters of the recording system were used: amplifiers sensitivity: 20 *μ*V/div; filters: 10–300 Hz; notch filters: off; plot time: 83 ms; and artefact reject threshold: 8% (for the amplifiers range ± 100 *μ*V) according to the local laboratory standards [15]. The response density (nV/degree^2^) and culmination time (ms) of the *P*1-wave in six rings were analysed.

The ERG recordings were obtained binocularly using Burian–Allen bipolar (Hansen Ophthalmic Development Lab, Coralville, Iowa, United States) with a ground reference electrode (Natus Europe GmbH, Planegg, Germany) placed on an earlobe. Pretesting preparation of the patient included instillation of 0.5% proxymetacaine as well as dilation of the pupils with 1% tropicamide and 10% phenylephrine hydrochloride. Then, the patient was placed in a dark room with eyes closed and covered with black googles for a 30-minute dark adaptation. The dark-adapted rod response was first obtained using dim white flash of intensity equal to 0.012 cd·s/m^2^; then, a stronger white flash of 1.6 cd·s/m^2^ triggered a rod-cone response. The same intensity of the stimulus was used for the induction of oscillatory potentials. The wave amplitude (peak-to-trough) and peak time (measured from the onset of the stimulus flash to the particular wave peak) in dark-adapted ERGs were analysed. The following parameters of the recording system were used: amplifier sensitivity: 10-20-50 *μ*V/div; filters: 0.3-500 Hz, with an exception for oscillatory potentials extraction in which 75-500-Hz bandwidth filters were used; notch filters: off; time base: 5 ms/div; and artefact reject threshold: 0 *μ*V. The detailed description of the ERG settings has been described elsewhere [14].

The occurrence of side effects was assessed with the use of Common Terminology Criteria for Adverse Events (CTCAE) taxonomy [16]. None of the subjects was lost to follow-up.

### 2.5. Quality of Life (QoL) Assessment

The NEI VFQ-25 questionnaire was distributed in an interviewer-administered format before Lin− cell injection and at 6, 9, and 12 months after the procedure. This questionnaire was designed to evaluate quality of life in patients with chronic eye diseases [17]. The findings of NEI VFQ-25 questionnaire were analysed as a total number of points.

### 2.6. Statistical Analysis

Statistical analysis was performed using Statistica version 13.3 (TIBCO Software Inc., California, USA) software. The Shapiro-Wilk test showed that distributions of most parameters were significantly different from normal distribution; therefore, nonparametric tests were used. To study the dynamics of changes, the Wilcoxon signed-rank test was performed to compare pretreatment conditions and both subjective and objective posttreatment conditions at all follow-up points separately. Similarly, Wilcoxon signed-rank test was used to compare parameters of treated eyes with parameters of FE. The correlation between the pretreatment condition and consecutive follow-up variables was tested using Spearman's rank correlation coefficients (Rs). To evaluate the difference between two subgroups, the Mann–Whitney *U* test was used. The significance level was set at a *p* value below 0.05. The results are presented as the mean ± standard deviation, unless stated otherwise for variables with strong deviation from normal distribution.

## 3. Results

### 3.1. Subjective Effects

The mean baseline BCDVA in eyes subjected to intravitreal therapy with Lin− cells was 14.5 ± 11.22, and it was 19.53 ± 11.94 in FEs. A significant improvement in BCDVA was observed from one month up to 12 months after the procedure (Table 2). One month after the procedure, patients read an average of 18 ± 13.59 (*p* = 0.045) letters on the ETDRS chart. At the 3-month visit, the result was 19.42 ± 14.35 letters (*p* = 0.027); at the 6-month visit, it was 18.96 ± 14.85 (*p* = 0.024), at the 9-month visit, it was 19.04 ± 14.63 (*p* = 0.012); and at the 12-month visit, it was 19.5 ± 14.42 (*p* = 0.019). Within the first 6 months after the procedure, 16 patients (53.33%) experienced BCDVA improvement. Accordingly, at 9-month and 12-month follow-up visits, BCVA increase was observed in 19 (63.33%) eyes and in 17 (56.67%) eyes, respectively. Since fluctuations in BCDVA in FEs during the follow-up were also observed, a comparative analysis of BCDVA changes in both eyes of the patients was performed. No significant differences between treated and fellow eyes at all follow-up time points were detected (Table 2). To identify the factors that might influence the BCDVA changes in eyes injected intravitreally with Lin− cells, we analysed the association between the baseline BCDVA and selected functional and morphological macular parameters. It was revealed that both BCDVA and contrast sensitivity in all follow-up examinations were positively correlated with the number of ETDRS letters read before administration of Lin− cells (Table 3). Moreover, the negative correlation with BCNVA was observed at most follow-up points (Table 3). These results suggest that patients who read more ETDRS letters before the cell therapy was implemented maintained their better BCDVA and BCNVA during the 12-month follow-up period. Interestingly, patients whose symptoms started less than 10 years previously gained 14 ± 10 letters, while those with longer disease durations gained only 2.86 ± 8.54 letters in the treated eye after the Lin− cell injection in the 12-month follow-up period (*p* = 0.021). Additionally, the baseline contrast sensitivity in group of eyes with RP diagnosis shorter than 10 years was higher than in eyes with a longer course of the disease. The median was 25 letters (IQR = 6.0) and 20 letters (IQR = 8.5), respectively (*p* = 0.005). Moreover, to evaluate the influence of baseline macular morphology on BCDVA presented after treatment, eyes subjected to Lin− cell therapy were divided into three subgroups based on the baseline OCT images. Accordingly, the macular edema was defined as the accumulation of serous fluid in the neurosensory retina causing central retinal thickening over 300 *μ*m involving or approaching the fovea. Contrary, the macular atrophy was characterized by features of incomplete retinal pigment epithelium, outer retinal atrophy with/or central retinal thinning less than 200 *μ*m. In cases of a lack of those features in the macular area in OCT, examination eye was considered as normal macula. Consequently, macular atrophy was detected in 11 eyes, macular oedema in 8 eyes, and normal macula in 11 cases. Interestingly, in the group with macular oedema, a significant negative correlation between baseline retinal volume in the macular region and BCDVA at 1 month (Rs = −0.886, *p* = 0.003), 6 months (Rs = −0.742, *p* = 0.035), and 9 months (Rs = −0.922, *p* = 0.001) after the intervention were observed. Accordingly, a significant negative correlation between baseline macular volumes and improvement in BCDVA in injected eyes (defined as difference in ETDRS numbers in follow-up timepoint and baseline BCDVA) at 1 month (Rs = −0.807, *p* = 0.015) and 6 months (Rs = −0.756, *p* = 0.029) of follow-up were found. Contrary, in the group with normal macula or macular atrophy, no significant correlations between baseline macular volume at 1-month (Rs = −0.033, *p* = 0.932; and Rs = −0.048, *p* = 0.91, respectively), 6-month (Rs = −0.109, *p* = 0.779; and Rs = −0.096, *p* = 0.821, respectively), 9-month (Rs = −0.109, *p* = 0.781; and Rs = +0.036, *p* = 0.932, respectively), and 12-month (Rs = −0.483, *p* = 0.187; and Rs = +0.036, *p* = 0.933, respectively) follow-up point were detected. Similarly, in both groups, no substantial correlations between baseline macular volume and improvement in BCDVA in injected eyes at any of the follow-up visits were noted. These findings indicate that better visual improvement in response to Lin− cell therapy was observed in eyes with less severe macular oedema.

Since visual field loss represents the leading problem in RP patients, an analysis of static perimetry parameters was performed. It was observed that the mean deviation (MD) values on 10-2 static perimetry improved significantly in treated eyes at the 12th month after the intervention (median = −19.35, IQR = 11.45) compared to baseline values (median = −21.64, IQR = 15.33; *p* = 0.032). To eliminate the possibility that this change was an incidental fluctuation, a comparative analysis of MD values in both eyes was performed. The MD improved significantly in the treated eyes compared to fellow eyes (median = 0.55, IQR = 1.78 for treated eyes and median = 0.03, IQR = 1.72 for FE, *p* = 0.017) 12 months after the procedure. No significant differences in 30-2 (W-W) static perimetry parameters were detected after the implementation of cell therapy in RP eyes.

In the course of intravitreal Lin− cell treatment in RP eyes, no significant changes in contrast sensitivity or BCNVA were detected.

### 3.2. Objective Effects

The limited response of rods in objective electrophysiological tests was consistent with an RP diagnosis. To assess the response of the central retina in the macular area as an effect of autologous Lin− cell intravitreal injection, mfERG was performed. We focused on the evaluation of cone system function detected in the first two rings of mfERG. The baseline mean response density of the *P*1-wave amplitude in the first ring was 43.22 ± 25.75 nV/degree^2^ in treated eyes and 55.99 ± 37.16 nV/degree^2^ in FEs. The subsequent analysis of *P*1-wave amplitudes in the first ring in the treated eyes showed significant improvement compared with pretreatment values in most follow-up examinations (Table 4, Figures 1(g)–1(i)). In contrast, in FEs, a significant decrease at most follow-up points was noted. Thus, an analysis of the differences between treated eyes and FEs was performed. Importantly, the response densities of *P*1-wave amplitudes in the first ring were significantly higher in treated eyes than in FEs at all follow-up points (Table 4).

Moreover, the response densities of the *P*1-wave in the first ring showed a positive correlation with the baseline values of this parameter at all follow-up time points, indicating that the higher the value was before the Lin− cell intravitreal injection, the higher the response during follow-up. Interestingly, 12 months after the procedure, the response densities of the *P*1-wave in the first ring were negatively correlated with BCNVA at this follow-up point (Rs -0.41, *p* = 0.034). This indicates that better cone system bioelectrical function corresponded with higher BCNVA in eyes subjected to Lin− cell therapy. No statistically significant differences in the response density of the *P*1-wave in ring 2 of the treated eyes were detected. Readings of mfERG peripherally to the R2 ring corresponding with the area outside of parafoveal region were nondiagnostic; thus, they were not included in the analysis.

The differences in the culmination time of the *P*1-wave in both rings of the treated eyes and FEs were not statistically significant at the majority of follow-up points. Similarly, on PERG, no statistically significant changes in the amplitude and peak time of the P50 wave or the amplitude of waves in the treated eyes and FEs were detected.

No significant differences in the ERG, central retinal thickness, or choroidal volume after the implementation of cell therapy in RP eyes were detected.

### 3.3. Impact of Cell Therapy on Quality of Life and Safety Issues of Lin− Cell Intravitreal Injection

**Figure 2:**
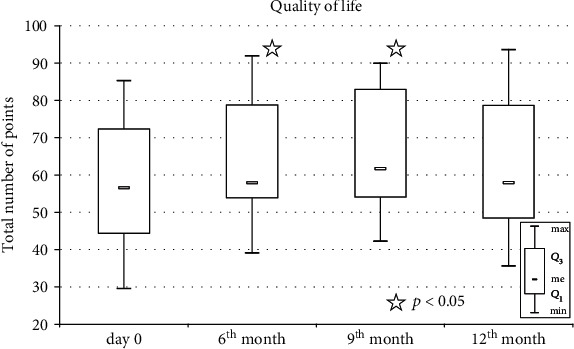
Boxplot representing NEI VFQ-25 quality of life score measured on day zero, six, nine, and twelve months after intravitreal administration of bone marrow-derived lineage-negative cells. The significance of the changes between baseline and follow-up timepoints was assessed using Wilcoxon signed-rank test. Whiskers range from 10th to 90th percentile. The square inside the box indicates the median. ∗*p* < 0.05.

To evaluate the influence of Lin− cell intravitreal injection on patients' daily activity, the analysis of a standardized QoL questionnaire was performed. The mean number of baseline points in the QoL questionnaire was 57.85 ± 15.95 and improved significantly to 64.54 ± 15.74 at the 6-month follow-up visit (*p* = 0.003), to 65.84 ± 15.7 at the 9-month visit (*p* = 0.001), and to 62.04 ± 17.35 at the 12-month follow-up visit (*p* = 0.063) (Figure 2). Moreover, the baseline QoL score showed a significant positive correlation with values identified on the consecutive follow-up examinations, indicating that patients with higher QoL prior to the Lin− cell intravitreal injection maintained their higher scores during the 12-month follow-up period.

Additionally, an analysis of the influence of selective ophthalmological parameters on QoL was performed. As a result, a positive correlation between QoL count and 10-2 MD values in treated eyes on corresponding visits was observed (Rs = 0.445, *p* = 0.02; Rs = 0.569, *p* = 0.003; Rs = 0.542, *p* = 0.004; 6 months, 9 months, and 12 months after the implementation of cell treatment, respectively). Similarly, 12 months after the procedure, a positive correlation between the corresponding MD values in 30-2 automated perimetry and QoL count was observed (Rs = 0.548, *p* = 0.005). This may indicate that patients with better perimetry findings in the course of the treatment declared a better QoL.

Significant negative correlations between the subjective rate of progression and QoL 6 and 9 months after the intervention were detected (Rs = −0.421, *p* = 0.026; and Rs = −0.519, *p* = 0.008, respectively).

The detailed assessment of both anterior and posterior segment of the eye as well as globe sonography did not reveal any features of local inflammation or uveitis. Additionally, the IOP analysis excluded any early spikes and confirmed stable values maintained till the 12-month follow-up visit. However, in two cases, local tractional retinal detachment secondary to vitreoretinal proliferation was detected 3 months and 12 months after the treatment. Consequently, pars plana vitrectomy with C3F8 endotamponade was performed. As a result, complete retinal attachment was achieved with no impact on final visual acuity. Those patients corresponded to the third grade of CTCAEs due to the indicated operative intervention. Additionally, in one individual, localized distant peripheral tractional detachment of the retina in the eye after implemented cell therapy was detected but did not require further treatment. That case was consistent with asymptomatic retinal detachment classified as a first-grade CTCAE.

## 4. Discussion

The main goal of this study was to assess the biological effects of single intravitreal Lin− cell injection in patients with retinitis pigmentosa. Notably, it is the first scientific report documenting the results of a clinical trial with the use of an autologous bone marrow-derived Lin− cell population in RP patients. Although in 3 treated eyes, retinal detachment was noted, due to careful monitoring and early vitrectomy procedure, no permanent damage to retinal morphology and function was observed. Thus, we concluded that the study found no seminal clinical adverse effects of this procedure, which further confirms that bone marrow is a feasible source of cell populations for intravitreal procedures. Remarkably, we observed that RP patients responded to this innovative transplantation procedure since their bioelectrical macular response improved significantly compared to FEs and was maintained for a 12-month period of time. In our previous studies, we observed that the beneficial effect of Lin− cell therapy was by virtue of neurotrophic activity due to the release of an abundant number of trophic factors with neuroprotective features [18]. The previous clinical studies of our team with experimental Lin− cell therapy for neurodegenerative disease showed that medical improvement was not rapid or short term. For example, intrathecal Lin− cell transplantation in amyotrophic lateral sclerosis (ALS) patients induced the best medical results approximately four to eight weeks after cell administration and was longitudinally maintained for at least 16 weeks [19, 20]. Our preclinical data also demonstrated that transplanted Lin− cells could exert a neuroprotective function against acute retinal injury. This effect was considerably associated with the ability of the Lin− cell population to express neurotrophic factors with antiapoptotic properties [9]. Our group observed that intravitreal administration of bone marrow-derived Lin− cells in murine eyes after acute retinal injury led to the integration of the Lin− cells in the outer retinal layers, thereby improving the morphological retinal structure on OCT analysis and inducing molecular changes such as the downregulation of proapoptotic signalling pathways and the induction of neuroregeneration, partially through the secretion of neurotrophic factors, especially BDNF. Interestingly, intracoronary infusion of Lin− cells in patients with acute myocardial infarction was responsible for an increase in the plasma levels of several angiogenic factors, such as vascular endothelial growth factor, angiopoietin-1, basic fibroblast growth factor, and platelet-derived growth factor in parallel to increased BDNF production, indicating that Lin− cells are also a valuable source of angiogenic trophic factors with systemic activity [21].

It seems reasonable that cell therapy with neuroprotective activity creates a very promising therapeutic option for chronic RP, regardless of the genetic inheritance in RP. This potential novel treatment would especially be available for a wider range of individuals with RP than gene therapy. In this study, we observed functional retinal improvement as an increase in BCDVA, after the Lin− cell intravitreal injection in RP patients. This result was subsequently corroborated by a significant improvement in mfERG detected in the first (foveal) ring. This indicates the protective role of Lin− cells for cone function in retinas affected by RP. These observations support the hypothesis of Otani et al. [22], who proposed, based on their preclinical research, that photoreceptor decline in RP may be a result of vascular atrophy providing a decreased metabolic supply for degenerating retinal cells (9). Moreover, Otani et al. [22] observed that the improved function of cones is possibly due to the stabilization of retinal blood vessels in all three vascular plexuses. Likewise, it has been demonstrated that BDNF also stimulates the formation of new vessels through a local increase of VEGF concentration, suggesting that local regional delivery of BDNF may, in parallel, provide a probable novel mechanism for inducing neoangiogenesis resulting in a decrease of cone photoreceptor degeneration [22].

Recently, many preliminary preclinical and clinical trials have shown good tolerability and long-term effects of intravitreally transplanted therapeutic stem and progenitor cells. For example, after a subretinal transplantation of cultured human foetal-derived retinal progenitor cells in 8 patients with advanced retinitis pigmentosa, a moderate improvement in BCDVA was observed between 2 and 6 months after the treatment in 5 out of 8 individuals. In 3 individuals, BCDVA was stable even at the 12-month follow-up visit, while at the 24-month visit, 1 eye still presented improvement. The nature of this mechanism was not tested in detail, and the authors analysed only the incorporation of SPCs into the outer nuclear layer. They revealed with OCT that foetal photoreceptors increased the thickness of the outer nuclear layer due to putative cell migration. Although no immune adverse effects were observed, this source of SPCs for therapy may raise ethical controversies [23]. In another study, testing the feasibility of subretinal allogenic adipose tissue-derived mesenchymal stem cell injection, only 1 of 11 subjects with end-stage RP experienced BCVA improvement after cell transplantation. However, the same individual presented also an improvement in ERG and perimetry at the 6-month follow-up visit [24]. Park et al. reported that after intravitreal injection of autologous CD34+ bone marrow-derived cells, a single RP patient was observed for improvement in BCDVA and perimetry at a 6-month follow-up visit. However, there was no effect detected on ERG, mfERG, or microperimetry [25]. Another study based on 34 RP eyes injected with allogenic umbilical cord Wharton's jelly-derived MSCs into the subtenon space presented significant improvement in BCDVA and 24-2 visual field observed in the period between 6 and 12 months after the intervention [26]. Unlike our study, the significant improvement in BCDVA at the 12-month visit was detected only in autosomal dominant and autosomal recessive forms of RP [26]. Interestingly, in a preliminary study with 6 months of observation, Özmert and Arslan also detected a significant improvement in the *P*1 wave amplitudes and implicit times in rings 1-3 in mfERG [27]. Our results are consistent with this observation, confirming that the central retina is most affected by paracrine effects of transplanted cells. The possible mechanisms responsible for this action are activation of dormant photoreceptors in the predegeneration phase, Müller cell hypertrophy, or ectopic synaptogenesis. In contrast to our study, possibly due to enrolment of individuals with higher BCDVA, the authors were also able to prove a significant improvement in full-field flicker ERG at the 6-month visit.

Weiss and Levy [28] reported improvement of an average of 7.9 Snellen lines in 45.5% of 17 patients (33 eyes) with RP in 6 months of follow-up after combined administration routes of autologous bone marrow-derived total nucleated cells. Similarly, 80% of 10 eyes with Usher syndrome experienced an average improvement in vision of 36.4% on the logMAR scale following the same study protocol for 1 year [29]. Weiss and Levy also indicated that the disease duration or severity of retinal degeneration might influence the efficacy of cell therapy [28]. Similarly, in our present study, we observed that RP patients whose symptoms started less than 10 years previously experienced a greater improvement in BCDVA.

In another study, a transient effect in 32 individuals with RP was detected for 3 months after the intravenous infusion of allogenic umbilical cord-derived MSCs [30]. However, no statistically significant changes in BCDVA, visual field sensitivity, or VEP during the course of the treatment were observed in this study. This may indicate that most of the extraocular routes of therapeutic cell administration are ineffective in RP patients, confirming that cells administered intravenously are incapable of penetrating the blood–retinal barrier to become accumulated there and then eliminated [30]. Thus, we can expect that the potential and most beneficial effects of cellular therapy in RP individuals may be obtained only with intraocular (e.g., intravitreal) cell injections.

It is worth mentioning that several limitations of the reported studies must be pointed out (i.e., noncomparable individuals in parallel groups, different stages of retinal degeneration, no electrophysiological or perimetry evaluations, follow-up evaluations performed by several doctors and with different equipment, consequently doubtful repeatable lighting conditions, and BCDVA measurements on the less sensitive Snellen chart in comparison to the ETDRS chart) [28]. This considerable heterogeneity of the clinical studies and the groups in the same study limits the possibility of drawing a direct conclusion regarding the effects of different cell-based therapies in patients with RP. Notwithstanding, since these are preliminary studies on cellular therapies in RP, we can expect several discrepancies between the performed studies. Moreover, some fluctuations in eye function within the natural course of RP are observed. However, none of the previously reported studies analysed differences between treated eyes and FEs. To our knowledge, this is the first report that evaluated those changes.

It is worth highlighting that intraocular injection may cause some negative effects as well. In the present study, three cases of tractional retinal detachment were diagnosed. It should be highlighted that RP patients are particularly susceptible to vitreoretinal junction pathologies, such as atypical posterior vitreous detachment, posterior vitreoschisis, or formation of condensed fibre aggregates in the vitreous body [31]. Maintenance of RPE integrity during cell administration might help to prevent such a negative consequence of intraocular cell transplantation [24]. This may indicate the superiority of intravitreal over the subretinal injection and vitrectomy in the RP patients.

Importantly, Lin– cells are small in diameter and with low adhesiveness, thus, permitting them to enter deeply into the retinal layers. The abovementioned characteristics of Lin− cells specify their superiority over the other cell types tested clinically in RP to date, including very large in diameter and highly adhesive mesenchymal stem cells or poorly enriched mononuclear cells. Finally, the autologous origin of Lin− cells permits for the lack of an immunosuppressive strategy during life-long treatment of RP in contrast to allogenic transplantation of foetal SPCs or genetically transduced adult photoreceptors. This highlights the meaning of preclinical studies based on animal models in search for an optimal protocol and administration regimen of cells prior to clinical trials. In our opinion, the intravitreal administration of Lin− cells is optimal and has high potency to induce neuroprotection of the injured retina in preclinical studies [9].

The natural progressive course of the disease with anticipated vision loss suggests the possible impact of cell therapy on QoL in patients who undergo intravitreal cell injection. Siquiera et al. [32] reported a significant improvement in vision-related QoL, which was observed 3 months after the implementation of autologous bone marrow-derived SPCs compared to baseline values before transplantation. However, at the 12-month visit, a deterioration of the QoL score was detected in this study. Interestingly, a positive correlation of QoL with macular sensitivity on microperimetry was described [33]. Accordingly, the QoL score analysis revealed a significant improvement at 3 months after the intravenous administration of umbilical cord-derived MSCs [30]. Moreover, a short-term improvement in BCDVA was also observed in this group [30]. In our study, we observed an improved QoL score for 9 months. Interestingly, we also observed a positive correlation between QoL scores and 10-2 MD and 30-2 values, demonstrating that higher QoL values are dependent on perimetry findings. However, the psychological characteristics of RP patients enrolled in the study could also have influenced the final results. We observed that RP patients with a higher baseline QoL presented better attitudes through the course of several follow-up visits. Moreover, a higher baseline subjective rate of RP progression resulted in lower QoL scores at 6 and 9 months after the therapeutic experimental intervention.

## 5. Conclusions

In summary, this is the first clinical study to evaluate the therapeutic activity of autologous bone marrow-derived lineage-negative cells administered intravitreally in a large and homogenous group of RP patients. We documented that Lin– cell-based therapy is safe and effective for a long period of 12 months, especially considering the improvement in BCDVA, MD of 10-2 perimetry, *P*1-wave response density in first ring mfERG, and QoL for a well-selected group of RP patients who have maintained good function of the foveal cones. Further clinical studies need to be conducted in the future to fully define the exact therapeutic roles of Lin– cells in biological treatment for RP. The results of this clinical experiment may help in the future to select patients for cell therapy to improve its efficacy and limit the risk of adverse effects.

## Figures and Tables

**Table 1:** Genetic characteristics of patients with retinitis pigmentosa.

Patient's number	Gene	Type of inheritance
1	Unknown	
2	*USH2A*	AR
3	*COL2A1*	AD
4	*IMPDH1*	AD
5	*PRPF31*	AD
6	*USH2A*	AR
7	*RS1*	X-linked
8	Unknown	
9	*USH2A*	AR
10	*IMPDH1*	AD
11	*USH2A*	AR
12	*PRPF31*	AD
13	*PRPF31*	AD
14	*CRB1*	AR
15	*PDE6B*	AR
16	*PRPH2*	AD
17	*IMPDH1*	AD
18	*EYS*	AR
19	*USH2A*	AR
20	*IMPDH1*	AD
21	Unknown	
22	Unknown	
23	*PRPF31*	AD
24	*USH2A*	AR
25	*PRPF3*	AD
26	*SEMA4A*	AD
27	*RP1*	AD
28	*RP1*	AD
29	*EYS*	AR
30	*USH2A*	AR

Abbreviations: AR: autosomal recessive; AD: autosomal dominant.

**Table 2:** The differences in best corrected distance visual acuity between the baseline and follow-up time point values in eyes treated with autologous Lin− cells and fellow eyes.

	Lin− cells	*p* value∗	Fellow eye	*p* value∗	*p* value∗∗
Baseline	14.5 ± 11.22		19.53 ± 11.94		
1 month	+3.5 (10)	0.045	+3 (8)	0.061	0.939
3 months	+3 (10)	0.027	+5 (8)	0.0002	0.137
6 months	+4 (10)	0.024	+4 (10)	0.01	0.917
9 months	+3.5 (9)	0.012	+5 (10)	0.002	0.42
12 months	+4 (10)	0.019	+2 (6)	0.004	0.742

∗Wilcoxon signed-rank test for comparison with pretreatment value. ∗∗Wilcoxon signed-rank test for comparison between treated eye vs. FE. Abbreviations: Lin− cells: lineage-negative cells; FE: fellow eye. Data were presented as median (interquartile range).

**Table 3:** Correlations between the BCDVA (ETDRS letters) and selected functional parameters in eyes treated with intravitreal injection of autologous Lin− cells.

	BCDVA (ETDRS letters)		BCNVA (Snellen)		Contrast sensitivity (number of letters on Pelli-Robson chart)	
	Rs	*p* value∗	Rs	*p* value∗∗	Rs	*p* value∗∗
Pretreatment	Ref.		−0.516	0.075	+0.427	0.033
1 month	+0.798	<0.001	−0.079	0.713	+0.563	0.003
3 months	+0.802	<0.001	−0.418	0.033	+0.379	0.056
6 months	+0.806	<0.001	−0.506	0.008	+0.537	0.005
9 months	+0.791	<0.001	−0.582	0.002	+0.514	0.007
12 months	+0.727	<0.001	−0.178	0.384	+0.407	0.048

Abbreviations: BCDVA: best corrected distance visual acuity; ETDRS: Early Treatment Diabetic Retinopathy Study; Lin− cells: lineage-negative cells; logMAR: Logarithm of the Minimum Angle of Resolution; BCNVA: best corrected near visual acuity; Rs: Spearman's rank correlation coefficient. ∗Spearman's rank correlation coefficient for evaluation of correlation with pretreatment BCDVA value. ∗∗Spearman's rank correlation coefficient for evaluation of correlation with pretreatment BCDVA and presented parameters in corresponding time points.

**Table 4:** Differences in mean response density (nV/degree^2^) of *P*1-wave in the first ring of mfERG between the baseline and follow-up time point values.

	Treated eye	*p* value∗	Fellow eye	*p* value∗	*p* value∗∗
Baseline	43.22 ± 25.75		55.99 ± 37.16		
1 month	+30.59 ± 0.14	<0.001	+0.4 ± 0.06	0.888	0.02
3 months	+29.89 ± 0	<0.001	−13.86 ± 0.01	0.179	<0.001
6 months	+10.25 ± 0.01	0.112	−21.61 ± 0	0.007	0.001
9 months	+8.58 ± 0.11	0.316	−20.91 ± 0	0.005	0.022
12 months	+11.18 ± 0.01	0.031	−18.79 ± 0.05	0.006	0.001

∗Wilcoxon signed-rank test for comparison with pretreatment value. ∗∗Wilcoxon signed-rank test for comparison between treated eye vs. FE. Abbreviations: mfERG: multifocal electroretinography; FE: fellow eye. Data were presented as mean ± SD.

## Data Availability

Data are available on request.
